# Argatroban for anticoagulation of a blood salvage system - an ex-vivo study

**DOI:** 10.1186/s12871-016-0204-3

**Published:** 2016-07-15

**Authors:** Martin Beiderlinden, Carsten Brau, Santo di Grazia, Michael Wehmeier, Tanja A. Treschan

**Affiliations:** 1 Klinik für Anästhesiologie, Marienhospital Osnabrück, Osnabrück, Germany; 2 Institut für Laboratoriumsmedizin, Marienhospital Osnabrück, Osnabrück, Germany; 3 KliPS Klinische Forschung – Patientennahe Studien, Klinik für Anästhesiologie, Universitätsklinik Düsseldorf, Heinrich-Heine-Universität Düsseldorf, Düsseldorf, Germany

**Keywords:** HIT, Patient blood management, Alternative anticoagulation, Blood salvage

## Abstract

**Background:**

Blood salvage systems help to minimize intraoperative transfusion of allogenic blood. So far no data is available on the use of argatroban for anticoagulation of such systems.

We conducted an ex-vivo trial to evaluate the effectiveness of three different argatroban doses as compared to heparin and to assess potential residual anticoagulant in the red cell concentrates.

**Methods:**

With ethical approval and individual informed consent, blood of 23 patients with contraindications for use of blood salvage systems during surgery was processed by the Continuous-Auto-Transfusion-System (C.A.T.S. ® Cell Saver System, Fresenius Kabi, Bad Homburg, Germany) using 5,50 or 250 mg of argatroban or 25.000 U of heparin in 1000 ml saline for anticoagulation of the system. Emergency and high-quality washing modes were applied in random order. Patency of the system and residual amount of anticoagulants in the re-transfusion bag were measured. The collected blood was not re-infused, but only used for analysis of hematocrit, heparin and argatroban concentrations.

**Results:**

Patency of the system was provided by all anticoagulants except for 3/8 cases with 5 mg of argatroban. Residual anticoagulant was found in 2/10 (20 %) heparin samples in two different patients (1 emergency and 1 high-quality washing) and in all argatroban samples. High quality washing eliminated 89–95 % and emergency washing 60–90 % of the initial argatroban concentration. Residual argatroban concentrations ranged from 55 ng ml^−1^ to 6810 ng ml^−1^, with initial argatroban concentrations of 5 and 250 mg, respectively.

**Conclusion:**

The C.A.T.S. does not reliably remove heparin and should therefore not be used in HIT patients. Anticoagulation with 50 and 250 mg argatroban, maintains the systems patency and is significantly removed during washing. In this ex-vivo study a concentration of 50 μg ml^−1^ argatroban provided the best ratio of system patency and residual argatroban concentration. Additional dose-finding studies with different blood salvage systems are needed to evaluate the optimal argatroban concentration.

## Background

Transfusion of allogenic blood carries the risk of severe complications, such as acute transfusion or hemolytic reactions or transfusion associated lung injury. It may also increase the risk of bacterial infections [1–3]. Due to these risks and increasing costs therapeutic alternatives to minimize allogenic transfusions are strongly recommended. The World Health Organisation recently highlighted the importance of such alternatives, typically referred to as patient blood management. One integral part of this “bundle” of measures is the use of blood salvage systems. These systems collect blood from the surgical site [4]: the salvaged blood is anticoagulated, washed, filtered and then concentrated for re-transfusion of “recycled” packed red cells. This method has been shown to reduce costs and the number of allogenic transfusions [5–7]. High dose anticoagulation within the blood salvage system is required to ensure its patency and to yield an adequate volume of blood for re-transfusion. Unfractionated heparin (UFH) is regarded the anticoagulant of choice for this application. However, there is only scarce evidence for the optimal anticoagulation regimen of UFH, as well as for the removal of UFH throughout different washing programs. Therefore, it is not well known, if residual unfractionated heparin is still present in the red cell concentrate [8, 9]. As the residual concentrations of unfractionated heparin can only be measured indirectly, there is some uncertainty about their potential systemic effects. Even little heparin amounts may be sufficient to cause complications in patients with antibodies against platelet factor 4-heparin-complexes as in patients with heparin-induced thrombocytopenia (HIT) [10]. HIT is a rare but severe complication of exposure to heparin with an overall incidence of 1 % in medical patients [11] and up to 10 % in patients with cardiac assist devices [12]. Although the incidence of the disease is low, it is frequent enough to be a clinically relevant problem, because HIT causes venous and arterial thromboembolic complications due to an up-regulated pro-coagulatory system [10]. In case of suspected HIT, all heparin exposure must be stopped immediately and strictly be avoided, while therapeutic anticoagulation with an alternative anticoagulant has to be initiated [13]. Argatroban, a direct thrombin inhibitor, has been approved for this specific indication and has shown to be safe and effective for anticoagulation of extracorporeal systems such as continuous renal replacement therapy and extracorporeal membrane oxygenation [14, 15].

In contrast, no data are available on the use of argatroban for anticoagulation of blood salvage systems. Patients with a history of HIT or with HIT suspect, who need to undergo surgery with a major risk of bleeding, should not be withheld the benefits of patient blood management, including blood salvage systems. Nevertheless, exposure to any concentration of heparin has to be prevented. For this purpose sodium citrate solutions may be used [16]. Alternatively, blood salvage systems could be anticoagulated with an alternative anticoagulant like argatroban in these specific patients. However, the use of argatroban for anticoagulation of blood salvage systems has not been studied yet.

Thus, we conducted a prospective randomized ex-vivo trial to evaluate the effectiveness of the anticoagulant effect of three different argatroban doses in comparison to a standard dose of unfractionated heparin and to assess potential residual anticoagulant in the washed red cell concentrate processed by the blood salvage system C.A.T.S.®.

## Methods

This ex-vivo study was performed in accordance with the Declaration of Helsinki in its current form after approval by the Ethics Committee of the Medizinische Hochschule Hannover (number 897/23.12.2010) and with patients’ written informed consent to donate their blood. Patients received oral and written information about the ex-vivo trial during pre-anesthetic consultation. Patients were eligible for donating their blood if they were planned to undergo major surgery with an expected intraoperative blood loss of more than 1 l and if they had contraindications for the use of a blood salvage system, such as malignancy or an infection at the surgical site. Exclusion criteria were age < 18 years, pregnancy, and surgery with planned use of a cell saver system. In this trial patients donated their blood to be treated and studied ex-vivo. Patients were not assigned to different treatment groups and no patient related health outcomes were studied. Thus, this research was not registered in a clinical trials database. The patients received nor anti-thrombotic treatment nor prophylaxis before start of operation.

### Blood salvage system and randomized anticoagulation

Blood of participants was collected intraoperatively from the surgical site and randomly assigned by sealed envelopes to be treated ex-vivo with one out of four anticoagulation study solutions for the blood salvage system, each prepared with 1000 ml of saline: 1) 25.000 U of unfractionated heparin (heparin-group), 2) 5 mg argatroban (argatroban 5 mg-group), 3) 50 mg argatroban (argatroban 50 mg-group) or 4) 250 mg argatroban (argatroban 250 mg-group). Blood was not re-transfused after processing.

In all four groups, blood was processed as follows: The reservoir of the Continuous-Auto-Transfusion-System (C.A.T.S.® Cell Saver System, Fresenius Kabi, Bad Homburg, Germany) was initially flushed with 100 ml of the study solution to moisture the internal filter. Upon start of surgery, a continuous infusion of the study solution of 100 ml h^−1^ was started via the routine surgical suction device in order to immediately anticoagulate the blood collected from the surgical site. To have comparable conditions in each group, the mixture of blood and study solution was then harvested into the reservoir of the blood salvage system until the collected volume was 600 ml. Then, from the reservoir of each patient two different washing programs were performed in random order: 1) emergency washing and 2) high-quality washing. Each washing program processed approximately 300 ml of collected blood. The main difference between the programs is a more intensive washing procedure using a sevenfold larger washing volume during high quality wash. In order to avoid interference with residual contents of the system after the first washing program, the connector of the reservoir, the “washing chamber” and the re-transfusion bag were exchanged prior to the second washing program.

After washing, the red cell concentrates were pumped into the re-infusion bag.

In all patients, samples for hematocrit (EDTA monovettes, E 772 G, 3,5 ml, Kabe Labortechnik, Nürnbrecht-Elsenroth, Germany), heparin and argatroban concentration (Sodium citrate monovettes, C 772 G, 4 ml Kabe Labortechnik, Nürnbrecht-Elsenroth, Germany) were taken from patients, the reservoir shortly prior to the start of the first washing program and from each of the two re-infusion bags after emergency and high quality washing.

Hematocrit was analyzed in our laboratory immediately using a Sysmex XN-2000 machine (Sysmex Germany, Norderstedt, Germany) whereas the citrated probes for argatroban and heparin concentration measurements were centrifuged (10 min at 1860 g) and the plasma stored at −80 °C for subsequent batch analyses.

Since heparin is a polymer consisting of sulfated glucose-amino-glucans of various chain lengths, the molecular size and molecular weight ranges widely. Therefore, a defined molar concentration cannot be measured directly rather than that plasma level of heparin can only be assessed indirectly via its pharmacological effect by monitoring the anti-Xa-activity. This method has a detection threshold value of < 0, 1 U ml^−1^ (Gerinnungspraxis Mannheim, Germany, Prof. Dr. C. E. Dempfle). Direct measurement of argatroban concentration was performed by high performance liquid chromatography and double mass spectrometry detection (Analytical Clinical Concepts GmbH; Leidersbach) [17].

### Sample size and statistics

For the question addressed in this trial, no data or preliminary results were available. Therefore, no formal sample size estimation had been performed. We prospectively aimed to treat blood from five patients with each anticoagulation study solution. In case of minor blood loss or clotting within the blood salvage system, the study could not be performed. Thus, patients were consecutively recruited until five cases per study solution could be completed.

Data are presented as median and range. Hematocrits were compared between the groups using univariate ANOVA. Calculations were done with SPSS Statistic Software Version 21. IBM.

## Results

In total 42 patients were eligible for the study and agreed to donate their blood collected from the surgical site for the purpose of this trial. In 19 patients blood loss was minor and thus no blood was processed. In three patients, blood that had been treated with 5 mg argatroban in 1000 ml saline clotted in the blood salvage system and therefore washing could not be performed. As a result, blood from 20 patients (age 70 (44–83) years, 12 male, 8 female) was processed by the blood salvage system, 5 patients per anticoagulatory regimen, each undergoing emergency and high quality wash. These patients underwent major surgery due to malignancies. As no opportunity for irradiation of blood prior to re-transfusion is available in our hospital, auto-transfusion of blood from the blood salvage system was contraindicated. Patients underwent esophagectomy (*n* = 5), pancreaticoduodenectomy (*n* = 7), liver resection (*n* = 3), gastrectomy (*n* = 1), radical hysterectomy (*n* = 1), rectum amputation (*n* = 1), nephrectomy (*n* = 1) and osteosynthesis for a pathologic femur fracture (*n* = 1). Patients preoperative laboratory measurements did not differ between groups (Table 1).

**Table 1:** Preoperative coagulation values

	Heparin	Argatroban	Argatroban	Argatroban	*p*
	25.000 IE	5 mg	50 mg	250 mg	
Hemoglobin (mg/dl)	11 [8–12]	11 [8–13]	12 [10–13]	10 [6–16]	0.581
Hematokrit (%)	31 [27–38]	33 [26–38]	37 [30–38]	30 [17–44]	0.540
Quick (%)	103 [63–110]	103 [56–140]	90 [69–99]	85 [73–95]	0.256
aPTT (seconds)	28 [25–44]	31 [28–35]	33 [27–35]	31 [28–33]	0.667
Fibrinogen (mg/dl)	262 [168–347]	272 [115–333]	379 [245–422]	300 [238–327]	0.158

### Heparin clearance

The study solution of 25.000 U of heparin in 1000 ml saline resulted in a median anti-Xa activity of 1,9 (0,4- 3,8 U ml^−1^) in the reservoir bag of the blood salvage system. This anti-Xa activity resulted in an effective anticoagulant state preventing the collected blood from clotting. After using the emergency wash program, heparin content in the red cell concentrate was below the detection threshold of 0,1 U ml^−1^ in four samples and 0,14 U ml^−1^ in one sample. After the high quality wash the anti-Xa activity in the red cell concentrate was below the detection threshold again in four samples and 0,27 U ml^−1^ in one sample. The two red cell concentrates containing residual heparin were not derived from the same patient. In total, 20 % (2 of 10) of red cell concentrates contained residual heparin activity, detectable by the anti-Xa activity test.

### Argatroban clearance

**Fig. 1:**
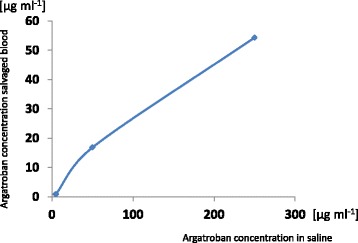
Argatroban concentration in saline and in the blood salvage system reservoir. Depicted are the median argatroban concentrations as measured in the blood salvage system reservoir versus the initial argatroban concentrations in saline used for anticoagulation of blood from the surgical field

Argatroban concentrations in the reservoir were lower than in the study solution, and increased linearly with increasing argatroban concentrations in the study solution (Fig. 1). Of note, washing of the collected blood could not be performed in three out of 8 cases (38 %) randomized to 5 mg argatroban in 1000 ml saline because of clotting within the blood salvage system.

In all other cases blood was successfully processed without clotting episodes.

**Fig. 2:**
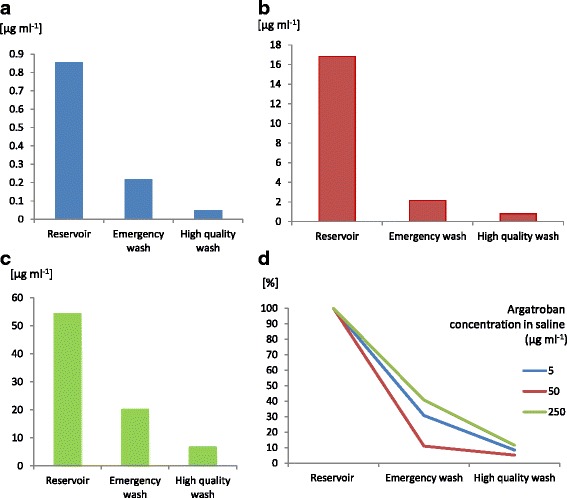
Argatroban concentration in the blood salvage system. Depicted are the median argatroban concentrations in salvaged blood reservoir (Reservoir) and in the red cell concentrates after emergency and high quality wash. Anticoagulation of the system with argatroban concentration in saline: **a**) 5 μg ml^−1^, **b**) 50 μg ml^−1^, or **c**) 250 μg ml^−1^; **d**) Comparison of the relative argatroban clearance after emergency or high quality wash - argatroban concentration in the reservoir was equalled to 100 %. The relative clearance did not differ significantly differences between the three groups

As shown in Fig. 2, residual argatroban concentrations were detected in all red cell concentrates, exhibiting lower values after high quality washing than after emergency washing. Emergency washing reduced the argatroban concentration to 10–40 % and the high quality wash reduced it to 5–11 % of the initial value in the reservoir. For both programs argatroban clearance was higher in the 50 mg than in the 5 or 250 mg study solution group (Fig. 2 panel d). However, there relative clearance did not differ significantly between the three groups.

After high quality washing, the lowest residual concentration was obtained with the 5 mg study solution: 55 (20–105) ng ml^−1^, while the highest residual concentrations were obtained with the 250 mg study solution: 6810 (3291–9916) ng ml^−1^.

### Hematocrits

Hematocrits were comparable in all patients prior to surgery: heparin-group 31 % (28–38 %), argatroban 5 mg-group 33 % (26–38 %), argatroban 50 mg-group 38 % (31–38 %), and argatroban 250 mg-group 30 % (18–45 %) (*p* = 0.66). In the reservoir bag, hematocrits remained comparable in all four groups: heparin-group 38 % (16-48 %), argatroban 5 mg-group 31 % (9–35 %), argatroban 50 mg-group 36 % (19-42 %), and argatroban 250 mg-group 31 % (24–48 %) (*p* = 0,58).

Hematocrits in the re-transfusion bags after both washing modes were approximately two-fold the initial patient hematocrits and similar after high quality or emergency washing, respectively:

Heparin-Group 65 % (52–72 %), 68 % (54–75 %), respectively, argatroban 5 mg-group 67 % (64–72 %), 64 % (51–70 %), argatroban 50 mg-group 68 % (51–74 %), 65 % (60–72 %), argatroban 250 mg-group 65 % (51–74 %), 68 % (56–77 %) (*p* = 0,88 for high quality washing and *p* = 0,87 for emergency washing).

## Discussion

We have studied the use of argatroban in the blood salvage system C.A.T.S.®. In this ex-vivo-study we tested the removal of argatroban and a standard heparin dose by two programs, “emergency wash” and “high quality wash”. Type and concentration of anticoagulation did not influence the general performance of the system, as hematocrits were comparable between groups. Our results provide additional information on the use of heparin and new information on the use of argatroban for anticoagulation within a blood salvage system:

First, although heparin was washed out from the majority of samples, there was residual heparin activity in 20 %. Thus, the heparin clearance within this system is not perfectly reliable. A finding that is consistent with previous reports [9, 18]. The interpretation of our findings has also to take into account that an absolute measurement of heparin concentration is not possible [19]. Therefore, we had to rely on the indirect detection of heparin by its anti-Xa-, and/or anti-IIa-activity. This indirect method cannot detect levels of heparin resulting in anti-Xa activities below 0,1 U ml^−1^. Therefore, we cannot exclude that heparin levels below this threshold value might have been present in our samples and may cause undesirable systemic effects. Consequently, we suggest refraining from using heparin anticoagulation for blood salvage systems in patients with suspected or proven HIT. Patients after HIT, i.e. in cases where HIT had been diagnosed previously, but where antibodies are no longer detectable at the time of surgery, a short period of exposure to heparin seems warranted [20].

Second, in patients with proven or suspected HIT intraoperative auto-transfusion is challenging. So far, lepirudin and danaparoid have been studied as anticoagulants for blood salvage systems, but at the moment both drugs are not broadly available [21, 22]. Regional citrate anticoagulation might also be employed [16, 23], but poses the risk of pH-disturbances. To test, whether argatroban is a suitable alternative we studied its use during two different washing programs. Emergency washing is intended to cope with acute and heavy blood loss. In this situation large volumes are collected in the reservoir in a short time and have to be processed immediately and fast. In order to achieve this, the volume of washing solution is 7 times less than for high quality washing. As a consequence, argatroban clearance was significantly better with high quality washing.

Due to lack of information on the use of argatroban with blood salvage systems, we used a very pragmatic approach and determined three different concentrations of argatroban for the study solutions. First we selected 50 mg argatroban, because its anticoagulatory potency correlates to 25.000 U of unfractionated heparin [24]. Then, a tenfold lower and a five times higher concentration was chosen hoping to approach potential limits of the lower and upper dosing range. Although we were finally able to process blood of five patients using the 5 mg argatroban study solution, the blood of three patients could not be washed because of clots in the reservoir. Therefore, a concentration of 5 μg ml^−1^ in the initial solution seems to be too low to reliably maintain the patency of the blood salvage system.

Red blood cell concentrate, yielded after using 50 μg ml^−1^ argatroban and high quality washing, contained 0,794 μg ml^−1^ residual argatroban. Thus, re-transfusion of 1000 ml red blood cell concentrate would theoretically result in an average argatroban “bolus dose” of approximately 794 μg. In a patient with a total blood volume of 6 l, this bolus would result in an argatroban plasma concentration of 132 ng ml^−1^. In comparison the plasma concentrations of argatroban in a steady state ranges between 300 and 600 ng ml^−1^.* Thus, such a single bolus from auto-transfusion would not be expected to cause clinically relevant anticoagulation. Nevertheless, clinicians would be reluctant to re-infuse any amount of an anticoagulant into a bleeding patient. Of note, after emergency washing, the residual argatroban was three times higher, and would thus lead to full anticoagulation. The study solution with 250 μg ml^−1^ argatroban resulted in an argatroban concentration in the red cell concentrate of 20 μg ml^−1^ after emergency wash. Depending on the volume of re-infused blood, this concentration has the potential to cause an excessive therapeutic anticoagulation resulting in a prolonged bleeding situation. Even with high quality wash the remaining argatroban concentration of 6,8 μg ml^−1^ might be harmful, if re-transfusion of larger volumes of red cell concentrates is necessary in a bleeding patient. Therefore, the use of argatroban concentrations as high as 250 μg ml^−1^ in the blood salvage system has to be heavily discouraged.

There is need for a dose finding study with argatroban concentrations below 50 μg ml^−1^ for anticoagulation of the salvaged blood.

This was an “ex-vivo”-study. Although, our design might come pretty close to the daily business we can only speculate about the effect of re-transfusion of red cell concentrates with residual argatroban concentrations in a heavily bleeding patient. Furthermore, for ethical reasons we used blood collected from patients with malignancies. We did that as to not withhold auto-transfusions from patients who would benefit from it. However, coagulation in patients with malignancies might differ from those without such a disease. We do not know, whether this might have influenced our results. We used only one blood salvage system. Therefore, we cannot rule out, if other systems do reliably wash out heparin and argatroban.

## Conclusion

Using the C.A.T.S.® heparin should not be used for anticoagulation of this blood salvage system in patients with suspected or proven HIT, as residual concentrations may occur. This has been affirmed for argatroban too. A concentration of 50 μg ml^−1^ argatroban is more effective than the 5 μg ml^−1^ for maintaining system patency, but seems to result in residual concentrations too high for re-transfusion. More information about the clinical consequences of auto-transfusion of red cell concentrates with residual argatroban concentrations and about alternative, lower doses are needed.

## Abbreviations

ANOVA, Analysis of variance; C.A.T.S., ontinuous-auto-transfusion-system; EDTA, Ethylendiamintetraacetat; HIT, heparin induced thrombocytopenia; IBM, International business machines; SPSS, Statistical package for the social sciences; UFH, Unfractionated heparin
